# Beyond tumor mutation burden: tumor neoantigen burden as a superior prognostic biomarker in resected gastric cancer

**DOI:** 10.3389/fimmu.2025.1722895

**Published:** 2026-01-05

**Authors:** Tingting Lu, Jingyi Cao, Peng Liu, Xiaoxin Pan, Yiming Shen, Jian Wang, Zhida Chen, Yi Liu, Fenglin Zhang, Haiya Zhang, Gan Zhang, Yi Wang, Ji Wan, Hongqing Xi

**Affiliations:** 1 Department of General Surgery, The First Medical Center, Chinese People's Libration Army (PLA) General Hospital, Beijing, China; 2 Shenzhen Neocurna Biotechnology Corporation, Shenzhen, China

**Keywords:** gastric cancer, prognosis, next-generation sequencing, tumor neoantigen burden, tumor mutation burden

## Abstract

**Introduction:**

Gastric cancer is one of the most diagnosed cancers and a major contributor to cancer-related death in China. Recent studies have demonstrated the failure of tumor mutational burden (TMB) in predictive accuracy. Here, we aimed to evaluate the prognostic value of tumor neoantigen burden (TNB) to predict the clinical outcome among patients undergoing gastric cancer resection.

**Methods:**

This study performs whole exome and transcriptome sequencing of tumor tissues of 85 gastric cancer patients who underwent resection surgery. The prognostic value of TMB and TNB in Chinese gastric cancer patients were evaluated, respectively.

**Results:**

In contrast to TMB, TNB achieved significance in predicting overall survival at both early (a median follow-up period of 23.5 month) and late (a median follow-up period of 35.5 month) timepoints (p-value<0.05), particularly with expanding 2-year and 3-year restricted mean survival time (RMST). According to the comparative enrichment and protein-protein interaction analysis of TNB- and TMB-associated genes, we found the upregulated TNB-associated genes were significantly enriched in hormone and nutrient sensing cascades, whereas their counterparts were predominantly linked to cytoskeletal development. In addition, we also noticed an increased infiltration of neutrophils in TNB-High group (p-value=0.04).

**Discussion:**

In summary, this study indicated that the prognosis of TNB-High patients was significantly better than their counterparts, which might be associated with impaired energy metabolism.

## 1 Introduction

As a major component of gastrointestinal cancer, stomach cancer has become the fifth incidence and fourth in mortality worldwide. Especially in Asian countries, stomach cancer remains the top three most prevalent malignancy in China (1), with an estimated 410,000 new cases were diagnosed every year (2). Due to the relatively lower sedation rate of gastroscopy, 80% of Chinese stomach cancer patients were diagnosed at an advanced stage (3), with the 5-year survival rate at 35.1% (4). Although surgery is considered as the pillar of its treatment strategies for stomach cancer patients at all ages, a significant number of patients experienced recurrence and metastasis even after curative re-section (5, 6). Given the complicated heterogeneity of gastric cancer, its prognosis can be influenced by many different factors including tumor burden, differentiation and staging, histological type, and lymph node metastases (7). Typically, a majority of the recurrences and tumor-related death occurred within 2 years of surgical resection (8–10). Therefore, more research is needed to identify a prognostic biomarker with enhanced sensitivity that is essential to stratify patients at high risk and guide treatment decisions. With the advent of targeted therapy for advanced gastric cancer, a punch of molecular biomarkers included HER2, FGFR2, CLDN18.2, and MET have been established (11). Moreover, as an important FDA-approved predictive biomarker for immunotherapy (12), PD-L1 diagnostic IHC assays are impaired by its dynamic expression, assay variability, and lack of standardized threshold (13). Recently, microsatellite instability (MSI) and/or DNA mismatch repair deficiency (dMMR) have emerged as the first tumor-type agnostic molecular biomarker (14). However, the prevalence of MSI-H/DMMR in advanced gastric cancer is fairly uncommon (less than 5%) (15). Another pan-cancer genomic biomarker, tumor mutational burden (TMB) measures the number of exonic somatic mutations, exhibiting predictive potential for immunotherapy efficacy (16) and becoming the only FDA-approved biomarker (17). Without an assessment of immunogenicity, TMB might be an imperfect surrogate biomarker for neoantigen-based immunotherapy (18). Previous work in non-small cell lung cancer (NSCLC) patients found significant difference in the number of oncogenic mutations and neoantigens produced (19), indicating the non-equivalency of TMB and neoantigen load. Noteworthy, the phase II study (KEYNOTE-158) demonstrated a positive association of high TMB (cutoff ≥10 muts/Mb) with progression-free survival but not overall survival (20). Again with the wide range for TMB and lack of universal TMB-High cutoff value (21), there is an urgent need for a clinically suitable biomarker to guide treatment selection.

High immunogenic neoantigens are widely acknowledged as the fundamental trigger to immunotherapy response. Tumor neoantigen burden (TNB) has recently emerged as an improved biomarker as a direct measurement of tumor immunogenicity. In another cohort study for patients with NSCLC, Rizvi et al. found that TNB, but not TMB, correlated to the efficacy of anti-PD-1 therapy (22). Although high TNB is expected to produce more neoantigens and thus contribute to an inflamed tumor microenvironment, its correlation to postoperative prognosis in gastric patients remains unclear.

In this study, we performed whole exome sequencing, and RNA sequencing from 85 resected gastric cancer patients. The gene mutation landscape, TMB and TNB indices, were systematically characterized. We demonstrated the prognostic value of TNB and its correlation with the survival outcome at two years postoperatively in gastric cancer patients, providing a novel insight into the clinical utility of TNB to guide precision medicine.

## 2 Methods

### 2.1 Patients and specimens

The study was approved by the Institutional Review Board and Ethics Committee of the Chinese PLA General Hospital (IRB number: S2021-503-01) and conducted in accordance with the Declaration of Helsinki. Clinical characteristics were curated from the electronic medical record of the hospital.

This study included a total of 85 patients diagnosed with gastric tumors who underwent surgical resections at the Chinese People’s Liberation Army (PLA) General Hospital (Beijing, China) between August 2021 and January 2022. The clinicopathological data for the 85 patients are summarized in **Table 1**. Paired tumor tissue samples and adjacent normal tissues were snap-frozen and stored at -80°C prior to DNA and RNA extraction. The primary outcome was cancer-related mortality. All patients were followed up in November 2023 and November 2024, respectively.

**Table 1:** Characteristics of study participants (n=85).

Variables	Frequency (N = 85)	Percentage (%)
Age		
Below 65	42	49.41
Above 65	43	50.59
Gender		
Female	22	25.88
Male	63	74.12
T		
T1	6	7.06
T2	9	10.59
T3	55	64.70
T4	15	17.65
N		
N0	27	31.77
N1	11	12.94
N2	16	18.82
N3	31	36.47
M		
M0	84	98.82
M1	1	1.18
Stage		
I	11	12.94
II	27	31.77
III	45	52.94
IV	2	2.35
Differentiation		
Poor	66	77.65
Well	19	22.35
Tumor Location		
Upper	41	47.67
Middle	10	11.63
Lower	27	31.40
Total	8	9.30
Resection		
R0	83	97.65
R1	2	2.35
Adjuvant Chemotherapy		
No Chemotherapy	9	10.59
Chemotherapy	76	89.41

### 2.2 Nucleic acid extraction and quality control

gDNA from frozen tumor tissue and adjacent normal tissue was extracted by MagPure FFPE DNA LQ Kit F (D6323-02F, Magen). Quantity and purity of gDNA was measured by Qubit 4.0 fluorometer (Invitrogen) and NanoDrop One (Thermo Scientific). Integrity of gDNA was measured by 4200 TapeStation system (Agilent Technologies).

Total RNA from frozen tumor tissue and adjacent normal tissue was extracted by TRIzon Reagent (CWBIO). Purity of total RNA was measured by NanoDrop One (Thermo Scientific). Quantity of total RNA was measured by Qubit 3.0 fluorometer (Invitrogen) using QubitTM RNA HS Assay Kit (Invitrogen). Integrity of total RNA was assessed by 4200 TapeStation system (Agilent Technologies) able to produce RNA Integrity Number (RIN). The tumor tissues were formalin-fixed, paraffin embedded (FFPE) and stained with hematoxylin and eosin. The tumor cell content was confirmed at least 10% by experienced pathologists.

### 2.3 Whole exome sequencing

According to Qubit quantification, a total of 50 ng of gDNA from each sample was taken for library construction using HP gDNA Library Preparation Kit (HaploX). After quality control and quantification by 4200 TapeStation system (Agilent Technologies) and Flex fluorometer (Invitrogen), a total of 500 ng library distributed between 280–350 bp was captured by KAPA HyperCapture Reagent kit (Roche) and sequenced on Illumina NovaSeq6000 platform (Illumina) at a depth greater than 150X.

### 2.4 RNA sequencing

Briefly, mRNA libraries were prepared using a range of total RNA input (1-4 μg). Sequencing libraries were constructed using VAHTS Universal V8 RNA-seq Library Prep Kit for Illumina (Vazyme) and purified by KAPA HyperPure Beads (Roche). Library concentration was determined by Qubit 3.0 fluorometer (Invitrogen) using QubitTM RNA HS Assay Kit (Invitrogen). The size distribution of RNA-Seq library was assessed by 4200 TapeStation system (Agilent Technologies). Libraries were pooled and sequenced by Illumina PE150 (paired-end 150 bp) on Illumina NovaSeq6000 platform (Illumina).

### 2.5 Detection of somatic mutations

The quality control was conducted by the following filter criteria: (1) Remove reads with low quality (below a mean of Q15); (2) Remove reads and with proportion of low-quality bases (N)>40%; (3) Remove reads with undetermined bases (N)>5; (4) Remove short reads (length<15). Clean data from WES and RNA-seq datasets were aligned to the GRCh38 reference genome with BWA and STAR to output the BAM records. Duplicate removal land Q value re-calibration were performed according to the GATK Best Practice (version 4.1) (23), generating BAM files. Short variants include single nucleotide, insertion and deletion (indel), gene fusion and microsatellite instability (MSI) were detected using GATK Mutect2. The high-confidence calls (labeled PASS) were further filtered using the following criteria: (i) Variants must have at least 4 supporting reads in the tumor sample; (ii) Variants with an allele frequency of at least 2%; (iii) Reads with low mapping quality (≤30) or mutated bases with low base quality (≤25) were excluded; (iv) The distances from the mutated base to the 5’ or 3’ ends of a supporting read must be less than 8 bp (the length of the barcode); (v) Variants with a minor allele frequency of less than 0.01 in the ExAC and gnomAD databases were retrieved. All VCF files were converted to MAF format using vc2maf and annotated using the ENSEMBL Variant Effect Predictor (version 100.0). Tumor mutational burden was reported as the total number of filtered variants per megabase of the coding region.

### 2.6 Copy number variation

Somatic Copy Number Alterations (SCNA) were analyzed using CNVkit (0.9.9). CNV status of a segment in tumor samples was calculated in a read depth-based estimation with a matched adjacent normal tissue. Regions of copy ratios greater than two-fold-change and less than 0.5-fold-change were defined as gain and loss of DNA segment for the downstream analyses respectively.

### 2.7 Mutational signature analysis

Six substitution subtypes (C > A, C > G, C > T, T > A, T > C, and T > G) were characterized for each sample. The profile of each signature was further expanded to 96 trinucleotide contexts by incorporating the adjacent 5’ and 3’ bases of the mutated site. The combination of 30 mutational signatures described in the COSMIC database (24) was determined by deconstructSigs (version 1.9.0) (25). The proportions of mutations assigned to each signature were quantified. Surv_cutpoint function in R package survminer was used to perform dichotomous grouping (high- or low-risk) of prognostic mutation signatures.

### 2.8 Tumor neoantigen burden

HLA typing from WES dataset was done for predicting Class I HLA genes. Quantitative analysis of RNA-seq dataset was conducted to determine gene expression levels of genes. Somatic mutations were annotated using Ensembl Variant Effect Predictor (VEP) to identify protein-changing variants. The frequency distribution of protein-changing variants in tumor samples was performed from WES and RNA-seq datasets. For neoantigen prediction, mutated peptide sequence was constructed by sliding window and the binding affinity between each peptide and HLA molecule was predicted using netMHCpan (26). Candidate neoantigen peptides was screened by the following criteria: (1) high HLA binding affinity; (2) expression of both mutated and paired ‘wild-type’ peptides can be detected from RNA-seq dataset. Tumor neoantigen burden (TNB) is defined by the number of neoantigens per megabase in the exonic region (neoantigens/Mb).

### 2.9 Differential gene expression profiling

R software version 4.01 was used for profiling differential expressed genes (DEGs) from the RNA-Seq data of the Beijing301 cohort. Patients were grouped into high and low groups by median TNB and TMB, respectively. The analysis was performed using package “EdgeR” with the cutoff criteria as |log2 fold change| > 1.5 and FDR<0.05. Volcano plots were created by the EnhancedVolcano package in R.

### 2.10 Gene ontology enrichment analysis

Gene ontology (GO) biological processes (BP) terms of specifically regulated DEGs were investigated using g:Profiler web server (27). BP terms presented with fewer than 10 genes or more than 500 genes were filtered out. Results with an adjusted P-value<0.05 based upon Bonferroni correction were accepted to indicate statistical significance. Heatmaps were plotted using SRplot (https://www.bioinformatics.com.cn/srplot) for data visualization (28).

### 2.11 Protein-protein interaction network analysis

The screened DEGs between two patient groups in terms of biomarkers (TNB, TMB) were uploaded to STRING (http://string.embl.de/) for PPI network construction. The PPI network of the screened DEGs with functional annotations obtained from STRING biological database were then visualized by Cytoscape software (version 3.10.3) (29).

### 2.12 Survival analyses

Overall survival (OS) was defined as the time from the date of surgery to the date of death or censorship at the last follow-up date. For the patients who died from causes unrelated to gastric cancer, OS was censored at their last known follow-up contact. Univariate, multivariate Cox regression and Kaplan–Meier analysis with a log-rank test in the R survival package was performed to assess the effects of different variables on survival. All statistical analyses were performed using R software version 4.1.3.

## 3 Results

### 3.1 General characteristics of study participants

The general clinical characteristics of 85 resected gastric cancer patients were listed in **Table 1**. Patient median age was 66 years, 74.12% were male and 25.88% were female. The median follow-up time was 35.5 months (0.9-44.4 months). Primary tumors were surgically removed from the following anatomical locations of the stomach according to the Japanese Classification of Gastric Carcinoma (30), including 27 (31.40%) from the upper part, 10 (11.63%) from the middle part, 41 (47.67%) from lower part, and 8 (9.30%) involved more than one part of the stomach. According to the TNM Staging Classification, 11 patients (12.94%) were stage I, 27 patients (31.77%) were stage II, 45 patients (52.94%) were stage III, and 2 patients (2.35%) were stage IV gastric cancer. Only one patient was presented with distant metastasis (M1), while all remaining patients identified as M0. In this cohort of patients, most patients showed R0 resection (85/87, 97.65%), while only two patients showed R1 resection. Nearly 90% of patients received adjuvant chemotherapy (76/85, 89.41%), including DS, SOX and XELOX regimens.

**Figure 1:**
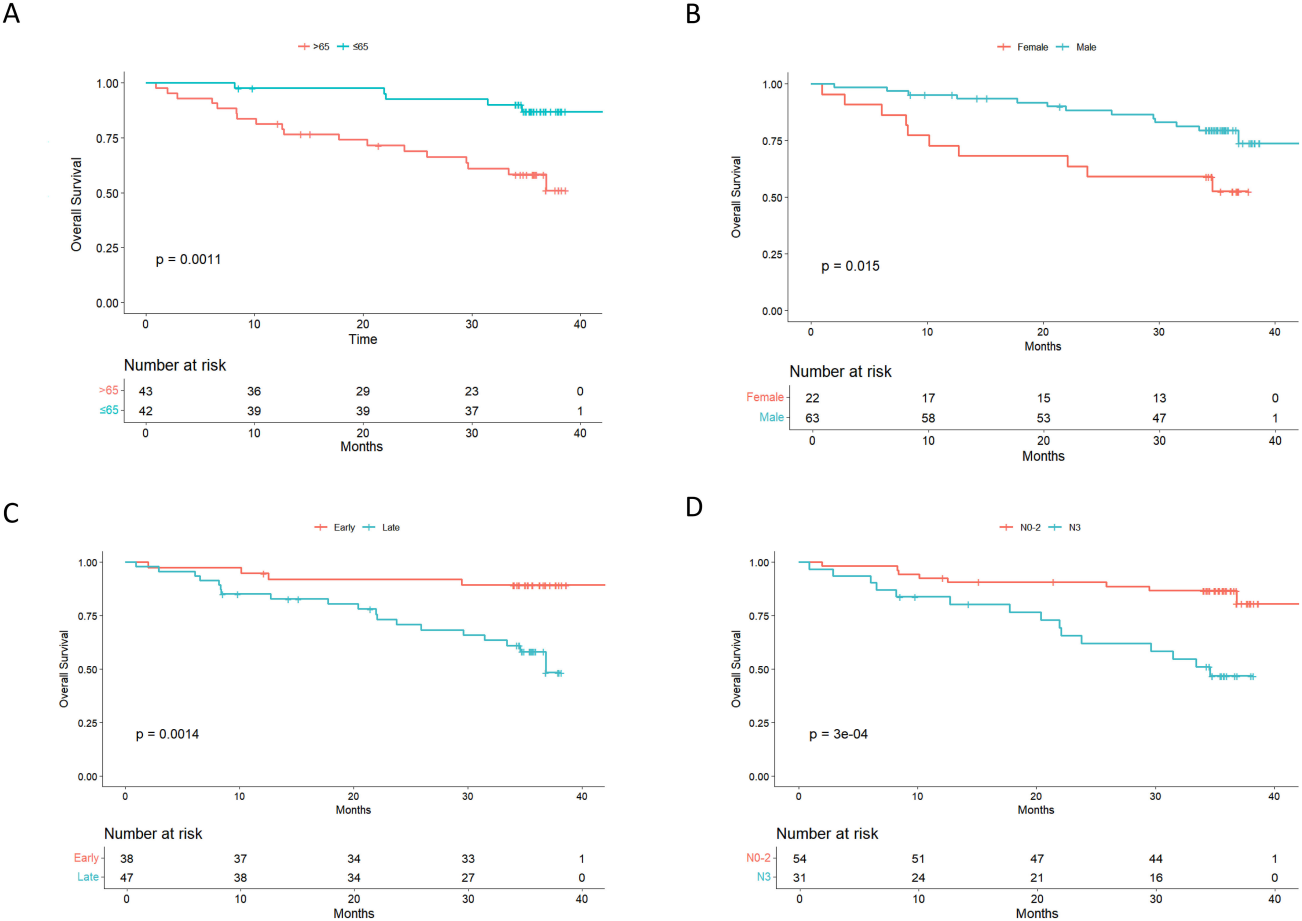
Kaplan-Meier estimates of overall survival (OS) according to age, gender, stage, and differentiation status, respectively. **(A)** OS stratified by age; **(B)** OS stratified by gender; **(C)** OS stratified by stage; **(D)** OS stratified by N Status; **(D)** OS stratified by N Status. Patients who died from non-cancer-related causes were censored, as indicated by the tick marks on the survival curve.

We performed a univariate analysis and demonstrated that age, sex, N status were prognostic factors (**Table 2**). Patients over the age of 65 (43/85, 50.59%) were associated with worse survival outcomes than younger patients (p-value=0.0011, **Figure 1A**). Approximately two thirds of patients were male, who experienced significantly longer overall survival after surgery than their female counterparts (p-value=0.015, **FSuure 1B**). We classified patients into early (I-II) and late (III-IV) stages and noted a strong correlation between tumor stage and OS (p-value=0.0014, **Figure 1C**). As nodal metastasis status varied across the cohort, patients with N0–2 exhibited an improved survival than patients with N3 (p-value<0.001, **Figure 1D**). We also compared the survival outcomes between patients with different pathological differentiation grades. Next, we compared the survival outcomes among patients with different grades of histologic differentiation by classifying patients into three distinct groups: high, moderate and low-grade differentiated tumors. Although patients with well/moderately differentiated gastric tumors had better overall survival but did not reach a statistical significance (p-value=0.46, **Figure 1C**). Specifically, there were no statistical differences observed in high versus moderate (p-value=0.40) and moderate versus low-grade tumors (p-value=0.98), and high versus low (p-value=0.40).

**Table 2:** Summary of univariate analysis for overall survival.

Variable	Group	n	HR (95% CI)	P value	Significance
Age	<65(Ref)	42	1.00 (Reference)		
	>65	43	4.50 (1.67-12.15)	0.003	**
Sex	Male (Ref)	63	1.00 (Reference)		
	Female	22	2.68 (1.17-6.12)	0.019	*
Stage	Early (Ref)	38	1.00 (Reference)		
	Late	47	4.90 (1.66-14.46)	0.004	**
N Status	N0-2 (Ref)	54	1.00 (Reference)		
	N3	31	4.32 (1.82-10.23)	0.001	***
Differentiation	Well (Ref)	19	1.00 (Reference)		
	Poor	66	2.08 (0.62-7.01)	0.236	
Chemotherapy	chemo (Ref)	76	1.00 (Reference)		
	no chemo	9	0.35 (0.05-2.57)	0.3	

### 3.2 Genomic landscape of somatic alterations

**Figure 2:**
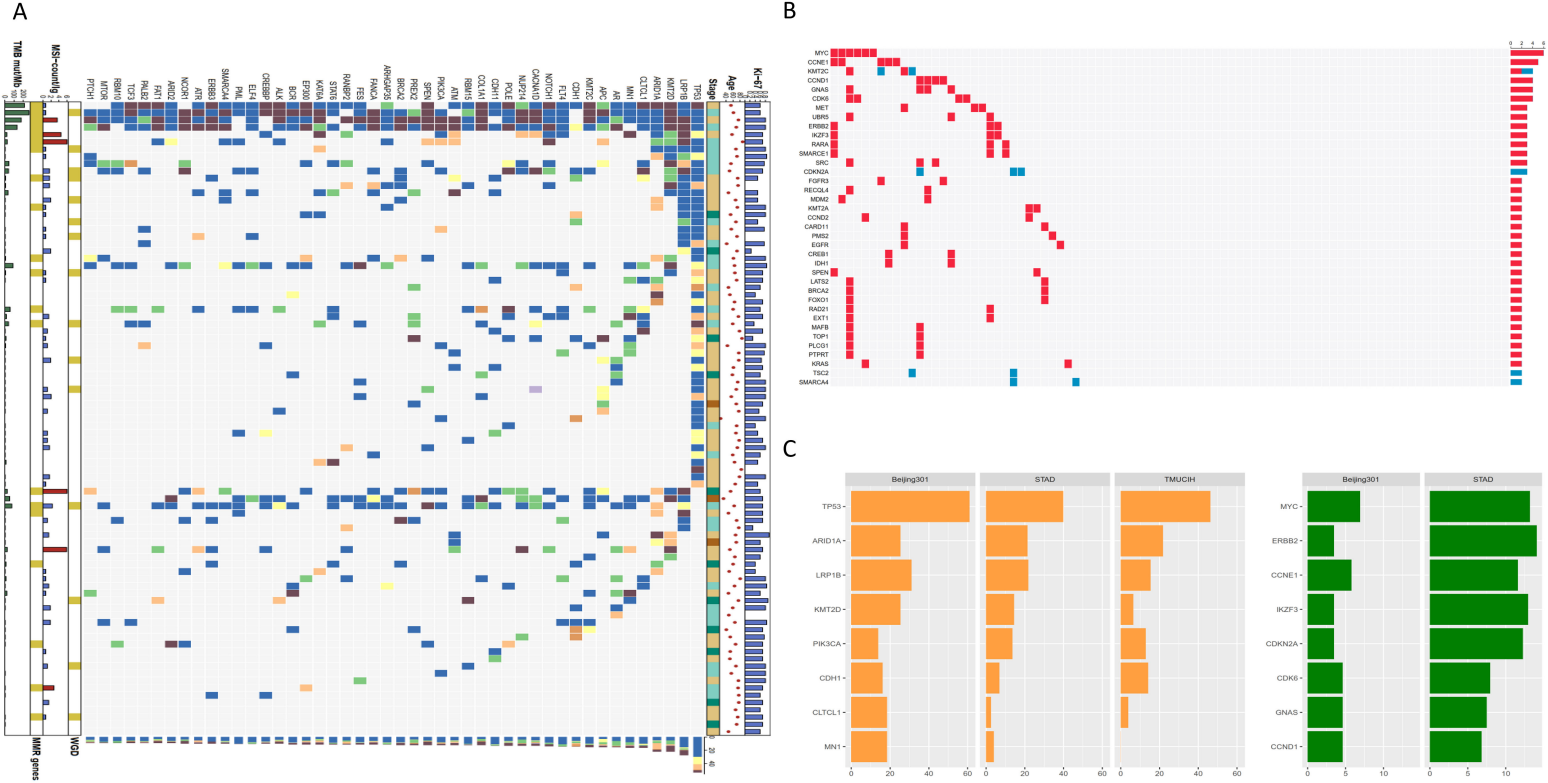
Comprehensive genomic profiling in gastric cancer. **(A)** The landscape of somatic mutations of 85 paired samples in Beijing301-GC cohort. The middle matrix shows the genomic alterations including missense, nonsense, and silent mutations, as well as alterative splicing and translation events by gene (row) and by sample (column). The top tracks show the median Ki-67 percentage and clinicopathological features including age and stage. The bottom tracks show the levels of microsatellite instability status and tumor mutation burden. The right histogram shows the number of predicted neoantigens and genomic alterations accumulated on 85 Chinese GC samples in each gene; **(B)** The copy number gain/loss occurrence in Beijing301-GC cohort (n=85) by gene (row) and by sample (column). The right histogram shows the total number of amplifications and deletions for selected genes per sample; **(C)** The left panel shows the comparison among Beijing301 (n=85), TCGA-STAD (n=441, queried from cBioPortal) and TMUCIH (n=206, queried from cBioPortal) GC datasets. The orange histogram shows the percentage of somatic mutations accumulated in each cohort for selected genes. The right panel shows the comparison between Beijing301 (n=85) and TCGA-STAD (n=441, queried from cBioPortal) datasets. The green histogram shows the percentage of copy number alterations accumulated in each cohort for selected genes.

To illustrate the somatic mutation landscape of gastric cancer, primary tumor and matched adjacent normal tissues of 85 patients were subject to whole exome sequencing. The read counts and average coverage obtained for each sample are presented in Data S1. Through an in-depth analysis, we identified a total of 1,980 single nucleotide variants (SNVs) and insertion-deletion (Indel) mutations across 399 genes using GATK Mutect2. The most frequently mutated gene was TP53, found in 60% (51/85) of the cases, followed by LRP1B with a mutation rate of 31.8% (27/85), KMT2D at 25.9% (22/85), ARID1A at 24.7% (21/85) and CLTCL1 at 18.8% (16/85) (**Figure 2A**).

We compared the high-frequency somatic driver gene mutations identified in this study (Beijing301) to two public datasets TCGA (STAD) and TMUCIH (31). The top three most frequently mutated driver genes, TP53, ARID1A, LRP1B were consistent across these three datasets. We discovered a higher rate of TP53 mutations in our Beijing301 cohort (60%) than in the TCGA (STAD, 39.7%) and TMUCIH (46.2%) cohorts, along with ARID1A, LRP1B, KMT2D, CDH1, CLTCL1, and MN1 (**Figure 2C**, left panel). Beijing301 and TMUCIH cohorts showed a comparable mutation rate across the most frequently mutated driver genes (**Figure 2C**, left panel).

Next, we explored the frequency distribution of copy number variations (CNVs). The most frequent CNVs were MYC gain (7%, 6/85) and CCNE1 gain (5.9%, 5/85) (**Figure 2B**). Majority of the high-frequency CNV genes were found to be overlapping between Beijing301 and TCGA (STAD) cohorts (**Figure 2C**, right panel), although relatively low incidence rate of CNVs detected in our study. Microsatellite instability (MSI) and Epstein-Barr virus (EBV) status were further investigated. Of the 85 patients, seven (8.24%) were MSI-H, and 6 (7.06%) were positive for EBV.

Considering the same race and ethnicity identities shared between these two cohorts, Beijing301 and TJMUCH (32), we attempted to repeat the clustering analysis and validated the distinct predictive performances of two most pre-dominant mutational signatures. The Kaplan-Meier survival analysis found that Signature 1 (p=0.031) indicated poor prognosis of gastric cancer patients, whereas Signature 6 was marginally associated (p=0.11) (**Supplementary Figure S1**).

### 3.3 Comparison between TMB and TNB in predicting overall survival

**Figure 3:**
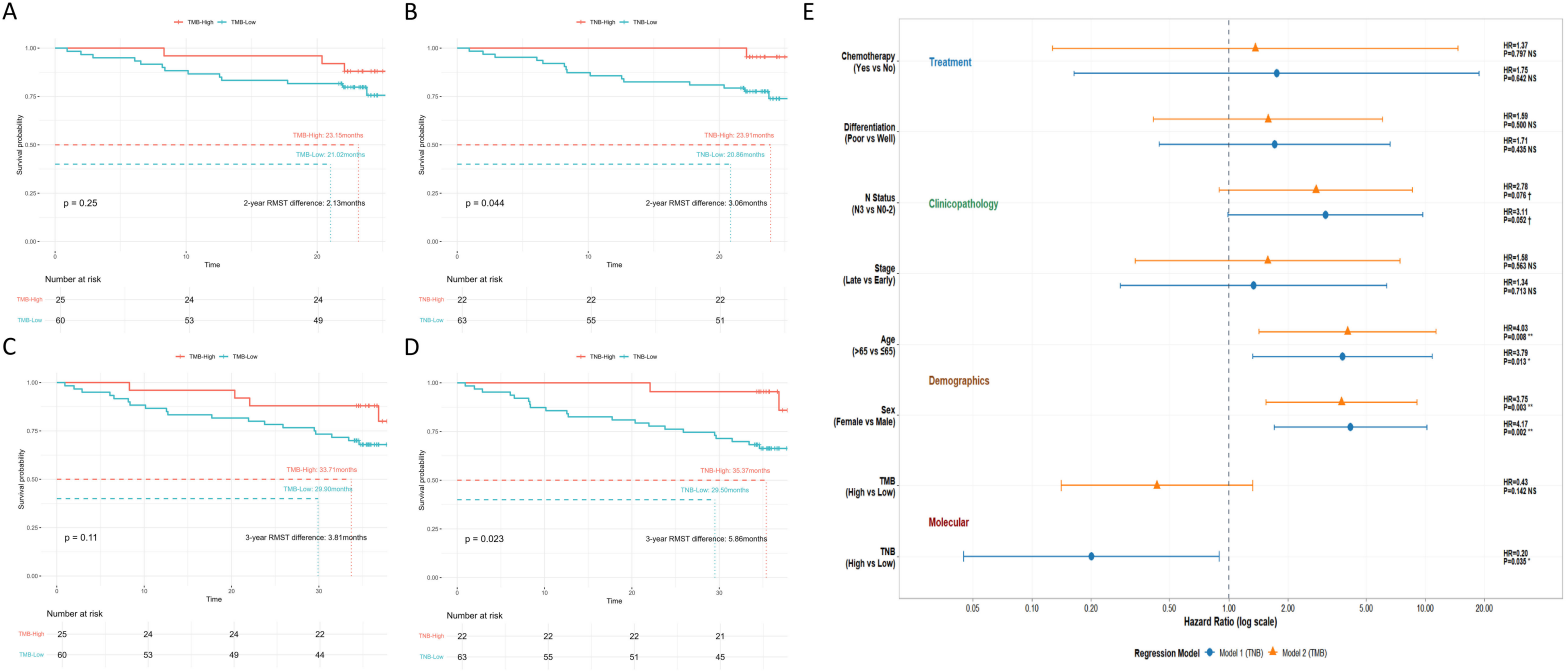
Impact of TMB and TNB status on overall survival. The impact of TMB status on overall survival after a median follow-up period of **(A)** 23.5 months (0.9-32.1 months), and **(B)** 35.5 months (0.9-44.4 months). The impact of TNB status on overall survival after a median follow-up period of **(C)** 23.5 months (0.9-32.1 months), and **(D)** 35.5 months (0.9-44.4 months). Patients who died from non-cancer-related causes were censored, as indicated by the tick marks on the survival curve. **(E)** Comparison of multivariate Cox regression models of TNB and TMB.

We analyzed and compared the impact of tumor mutation burden (TMB) and tumor neoantigen burden (TNB) on overall survival (OS). For this study, we defined the high TMB subgroup by using the top 30% of TMB (≥12 mut/Mb). A total of 25 patients (29.41%) had TMB-High tumors, and 60 patients (70.59%) were TMB-Low. Despite showing a numerical trend toward better overall survival, TMB failed to reach statistical significance at both timepoints (median follow-up period of 23.5 and 35.5 months) (**Figures 3A, B**).

Similar to a previous report (33), a low neoantigen load was observed in our cohort, thus we established a data-driven cutoff of 6 neoantigens/Mb using a stepwise gradient search strategy. Twenty-two patients (25.88%) were classified as TNB-High and 63 patients (74.12%) were TNB-Low. Strikingly, TNB exhibited enhanced power of distinguishing poor and good OS at a median follow-up period of 23.5 and 35.5 months, respectively (**Figures 3C, D**). To better evaluate the prognostic value of TMB and TNB, the restricted mean survival time (RMST) were calculated. Steeper increases in both the 2-year (3.06 vs 2.13 months) and 3-year (5.86 vs 3.81 months) RMST difference demonstrated the improved stratification power of TNB compared to TMB. Especially we noted a consistent survival advantage of TNB-High (2-year: +0.76 month; 3-year: 1.66 month) over TMB-High, while TNB-Low and TMB-Low trajectories nearly converged.

To better control the potential confounders and confirm the independent prognostic value of TNB and TMB, we performed multivariate Cox regression after adjusting for key clinical characteristics including age, sex, stage, N status, differentiation and adjuvant chemotherapy. Consistent with the univariate analysis, the multivariate Cox analysis confirmed that older age (>65), female sex were significant negative prognostic factors, with N3 status showing a trend towards significance (**Figure 3E**). Strikingly, TNB emerged as a significant independent factor for overall survival (p-value=0.035), while TMB showed no significant association (p-value>0.1).

### 3.4 Screening of DEGs according to different TNB and TMB levels

**Figure 4:**
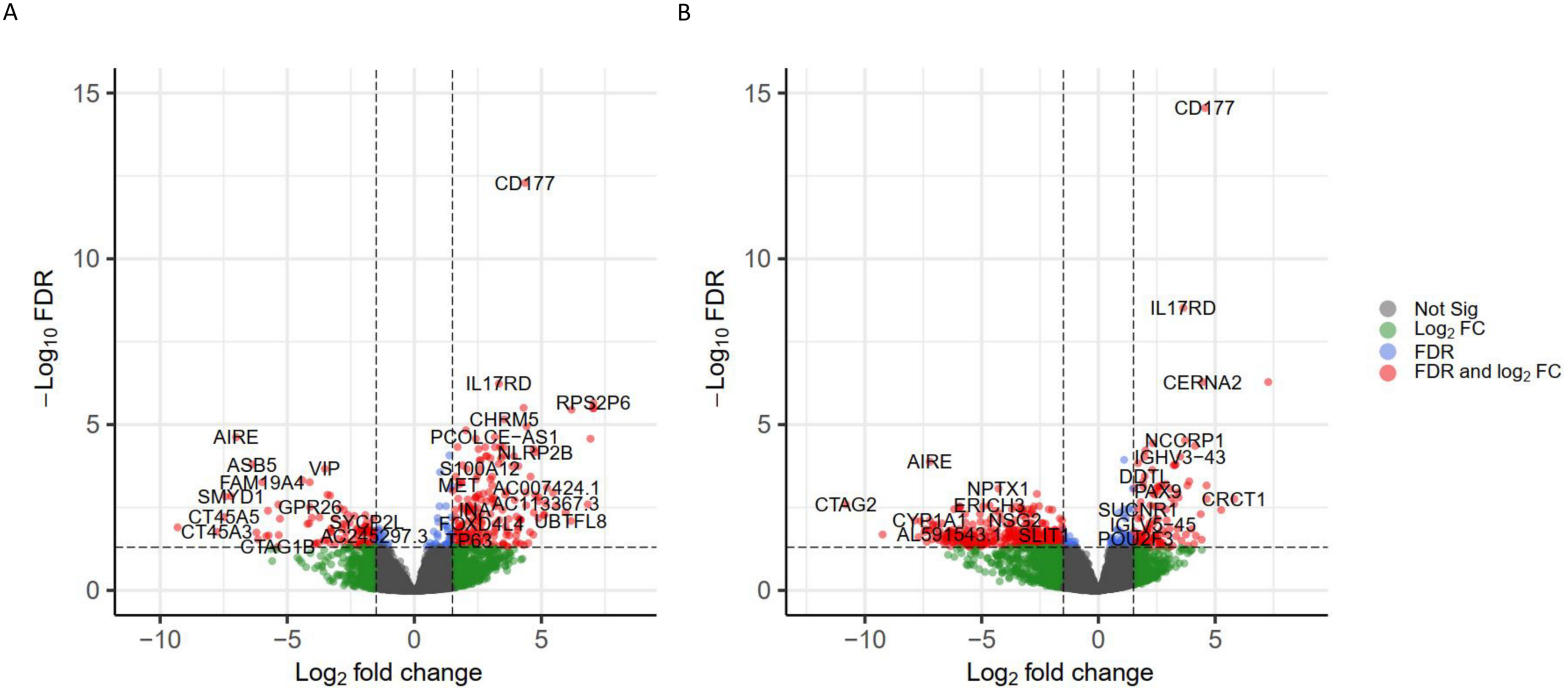
Identification of differential expressed genes in terms of TNB and TNB. The volcano plots of the differential expressed genes between **(A)** TNB-High and TNB-Low and **(B)** TMB-High and TMB-Low. Green dots represent |log2 fold change| greater than 1.5 but FDR>0.05. Blue dots represent |log2 fold change| less than 1.5 but FDR<0.05. Red dots represent genes with significant differential expression levels based on FDR and log2 fold-change, or not significant in bother terms (grey dots).

Next, we compared the mRNA expression profiles in terms of two biomarkers, TNB and TMB respectively. Differentially expressed genes were screened using the cut-off criteria as |log2 fold change| > 1.5 and FDR<0.05. A total of 546 DEGs were identified across the TNB-High and TNB-Low subgroups, in which 400 genes were upregulated, and 146 genes were downregulated (**Figure 4A**). Similarly, there were 303 DEGs between TMB-High and TMB-Low, among which 199 were upregulated and 104 were downregulated (**Figure 4B**). Not surprisingly, several cancer testis antigens (CTA) including CT45A1, CT45A3, CT45A5, CTAG1B and CTAG2 were highly expressed in TNB-High or TMB-High tumors. As a key mediator of appetite and energy balance, enhanced expression of leptin (LEP) and leptin receptor (LEPR) was detected only in TNB-High tumors. Additionally, the calcium-sensing synaptic vesicle protein, Synaptotagmin-4 (SYT4) expression increased only in TNB-High patients. We noted that CD177, IL17RD, TP63 and MET were highly expressed in both TNB-Low and TMB-Low patients. Increased expression of representative S100 proteins, including S100A7, S100A8 and S100A12 were observed in both TNB-Low and TMB-Low groups, whereas elevated expression of S100A9 was detected only in TNB-Low samples. The full lists of TNB- and TMB-associated DEGs are given in Data S2 and S3.

### 3.5 Functional enrichment and PPI analysis of DEGs

To delineate the distinct molecular function of the specific differentially expressed genes identified between high TNB and TMB subgroups, we performed Gene Ontology (GO) analysis using g:Profiler (**Supplementary Figure S2**). For upregulated TNB-associated DEGs, we found the strongest signal came from adult feeding behavior, following with regulation of response to food and nutrient levels, positive regulation of secretion, and adenylate cyclase-activating G protein-coupled receptor signaling pathway. For TMB-associated DEGs, myeloid leukocyte, granulocyte, and neutrophil migration were the most enriched BP terms.

Next, we further explored the interplay among the TNB- and TMB- associated DEGs, through reconstruction of their protein-protein interaction networks. For TNB-High specific DEGs, we obtained a Protein-Protein Interaction (PPI) network composed of 174 nodes and 323 edges. According to the node degrees calculated using Cytohubba, we found that NPY has the highest degree, following with a handful of hormone-encoding genes, such as OXT, GHRL, and LEP (**Supplementary Figure S3A**). PPI network of the TMB-associated DEGs was also constructed, including 113 nodes and 322 edges. Several immune-related genes, including IL6, IL1A, and CXCL8 were identified as key nodes (**Supplementary Figure S3B**).

**Figure 5:**
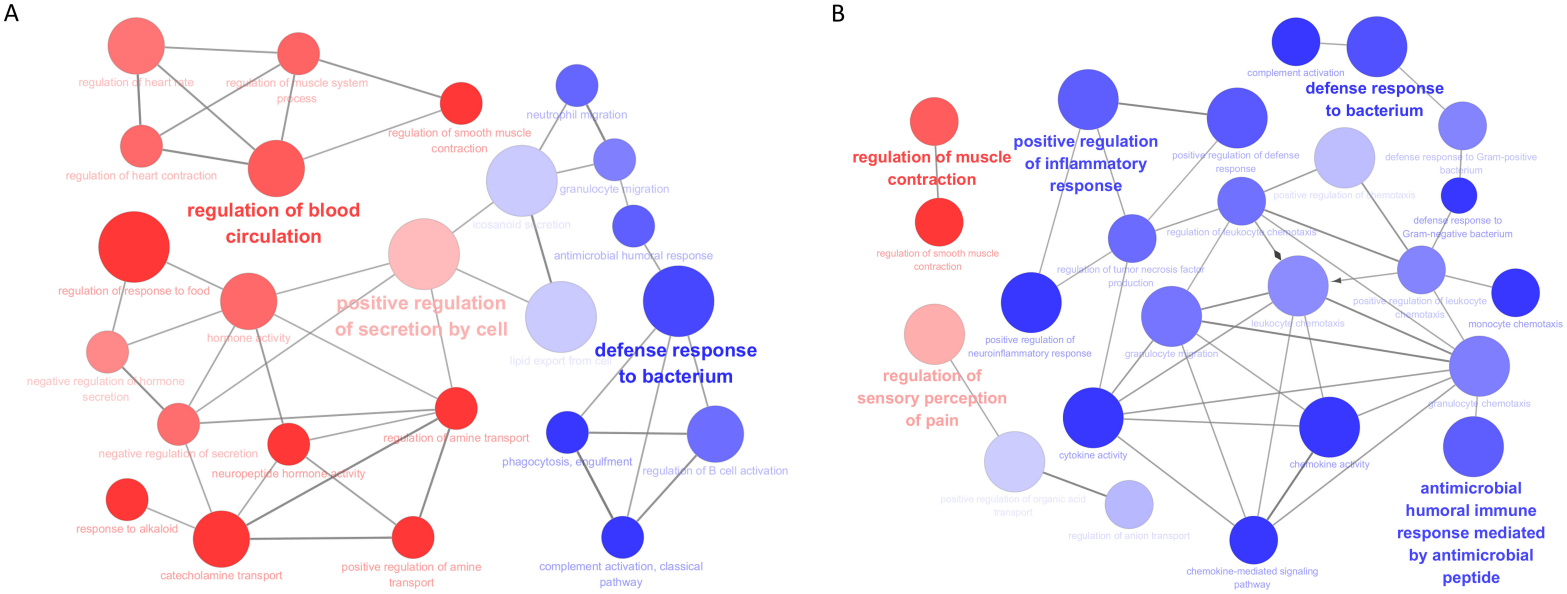
Functional annotation of TNB- and TMB-associated DEGs using ClueGO. **(A)** Enriched biological processes by TNB-associated DEGs. **(B)** Enriched biological processes by TMB-associated DEGs. Red dots represent upregulated DEGs and purple dots represent downregulated DEGs. The name of each group is given by the most representative term of the group. The nodes are grouped based on shared genes (kappa score).

To translate the specifically regulated genes into biological insights, we also performed the gene ontology analysis using ClueGO to identify cross-talk of their enriched pathways. For TNB-associated DEGs, the signaling pathways of upregulated genes were mainly enriched in regulation of blood circulation, positive regulation of secretion by cell, while the downregulated genes were related to defense response to bacterium. For TMB-associated DEGs, high expression of TMB correlated with regulation of muscle contraction and regulation of sensory perception of pain. Low-TMB was mainly associated with positive regulation of inflammatory response, defense response to bacterium, and antimicrobial humoral immune response (**Figure 5B**).

## 4 Discussion

Gastric cancer is often diagnosed at advanced stage in China, leading to an increased mortality rate. According a retrospective study, surgical resection of primary tumor seems to be beneficial for selected patients in prolonged OS (34). Postoperative survival in gastric cancer patients is influenced by the multitude of clinical characteristics, ranging from patient demographics to specific clinicopathological features (35). Initial univariate screening confirmed the classic prognostic factor, including age above 65 years, female sex, advanced stage, and N3 status, were significantly associated with reduced survival.

As a highly heterogeneous disease, many molecular and histological features were driven by genomic alterations occurred during the development of gastric cancer. In this study, we performed next generation sequencing from a total of 85 Chinese gastric cancer patients after surgical resection in primary tumor site. We integrated the landscape of somatic gene mutation including SNV, CNV and neoantigen load to understand these genomic heterogeneities.

As a newly established independent biomarker in cancer immunotherapy, tumor mutation burden has been proposed to predict patients’ response to immune checkpoint blockade. However, accumulated clinical trials and retrospective studies have indicated failures of TMB in prognosis very recently (21, 36, 37), attaching more attention to reconsider its clinical use. Challenges for TMB to be used in precise oncology settings include the following: (1) TMB is a measure of tumor mutation quantity, only some of these mutations can be processed into immunogenic neoantigens; (2) Due to the large variability of TMB across and within cancer types, it would be empirically hard to infer an optimal universal standard for TMB-High cutoff.

Contrary to TMB, TNB directly measures the tumor neoantigen load and thus reflects the tumor immunogenicity. With advances in computational neoantigen prediction, tumor neoantigen load has been used as an emerging predictive biomarker for immunotherapy response (38). In the present study with Chinese gastric cancer patients after primary tumor resection, we identified a TMB-High cutoff at 12 mut/Mb and TNB-High cutoff at 6 neoantigens/Mb. Noteworthy, the expanding RMST difference highlighted the superior prognostic potential of TNB-High. Furthermore, the multivariate Cox regression analysis provided compelling evidence for the prognostic value of TNB as an independent biomarker, even after adjusting for key demographic and clinicopathological variables. In contrast, TMB failed to demonstrate its independent prognostic power in our cohort. This divergence suggests that TNB may serve as a superior biomarker of postoperative survival, shedding light on future clinical application of TNB in precision oncology and patient stratification.

These results prompted us to further explore the involvement of TNB- and TMB-associated DEGs in biological process and signaling pathways. While both downregulated TNB- and TMB-associated DEGs converged on pathways related to defense response, their upregulated counterparts exhibited a stark functional divergence. In contrast to the TMB-associated cytoskeletal functions, TNB-upregulated genes were highly specific to hormone- and nutrient-sensing cascades. Given the critical role of energy metabolism reprogramming in tumor development (39), targeting the metabolic-hormonal axis disruption might introduce therapeutic benefits to improve the clinical outcome of TNB-High patients.

Still, there are some limitations that should be considered. First, the sample size (n=85) was relatively small and particularly in Chinese patients. Second but not least important, the retrospective design and the low event rate in the TNB-High group that hindered extended comprehensive analysis. Given the population-based-3-year postoperative survival for gastric cancer patients, telephone follow-up within a six-month period might be helpful to evaluate the time-varying sensitivity and accuracy of baseline prognostic biomarkers like TMB and TNB. Third, this cohort was relatively heterogenous, especially with marked clinical heterogeneity of gastric cancer according to tumor location and histopathological standpoint. Moreover, the samples were collected from the era before neoadjuvant immunotherapy, the prognostic value of neoantigen load needs to be further validated in both standard-of-care treatments and combined immunotherapy.

## 5 Conclusions

In conclusion, we conducted comprehensive genomic profiling of 85 Chinese gastric cancer patients after surgery and evaluated the prognostic value of TMB and TNB. Compared to TMB, high tumor neoantigen burden has shown promise as an emerging biomarker that shows superior capability in predicting survival outcomes, especially in a median follow-up period of two years. To our knowledge, this is the first attempt to provide evidence of the superior prognostic value of TNB in gastric cancer. Given this cohort predates its widespread clinical adoption of neoadjuvant/adjuvant immunotherapy, most of the patients received conventional chemotherapy. Although not in an immunotherapy context, nevertheless the finding provides evidence that supports further validation in prospective, larger-sample, and multi-center studies. With further validation, TNB might be a useful tool for risk stratification to guide prognostication in patients with gastric cancer. Moreover, the clinical significance of tumor neoantigen load across other cancer types and especially in the adjuvant settings needs to be further explored.

## Data Availability

The data presented in the study are deposited in the CNCB repository, accession number GVM001250.
