# Supplementary material for: Beyond tumor mutation burden: tumor neoantigen burden as a superior prognostic biomarker in resected gastric cancer
Source: Front Immunol. 2026 Jan 5;16:1722895. doi: 10.3389/fimmu.2025.1722895 (PMC12813098; doi:10.3389/fimmu.2025.1722895)
Supplement: Supplementary file 4 [file Table1.docx]

Supplementary Material

# Supplementary Data

**Supplementary Data S1.** The read counts and average coverage obtained for each sample.

**Supplementary Data S2.** The list of TNB-associatd DEGs.

**Supplementary Data S3.** The list of TMB-associated DEGs.

# Supplementary Figures


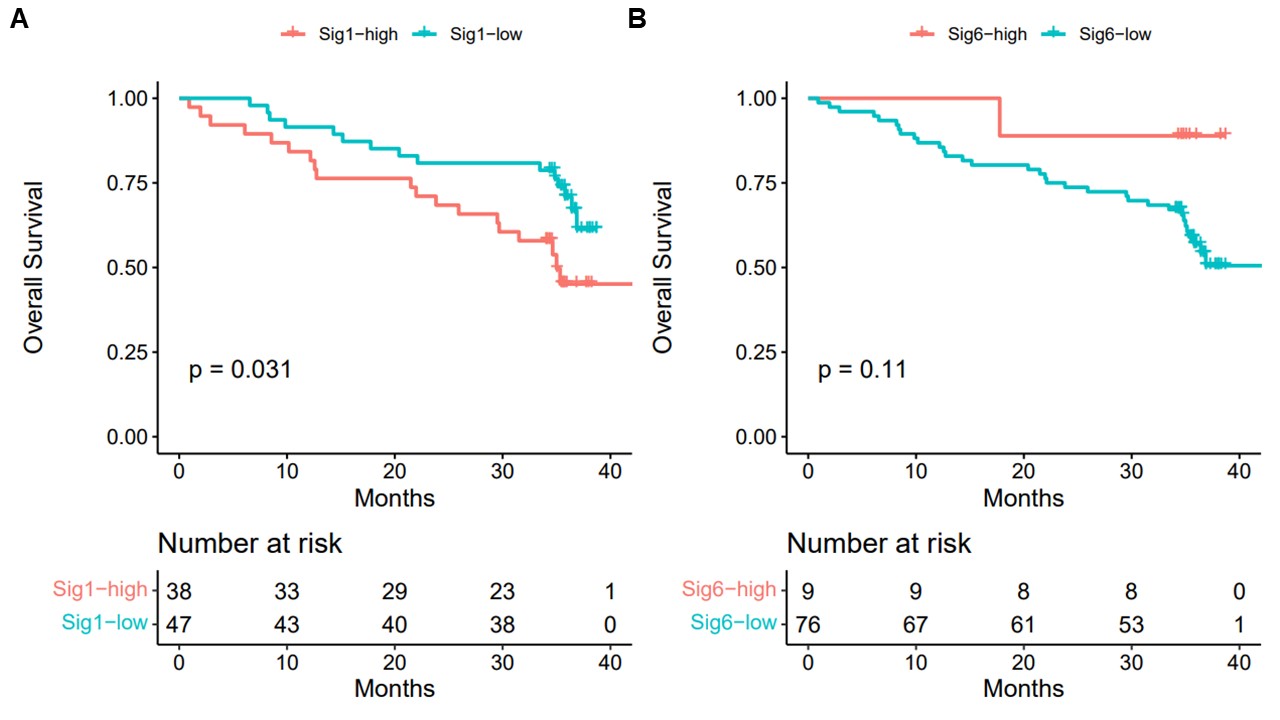


**Supplementary Figure 1.** Kaplan-Meier analysis was performed to analyze the impact of two pre-dominant mutational signatures. (A) OS stratified by Signature 1 (B) OS stratified by Signature 6.


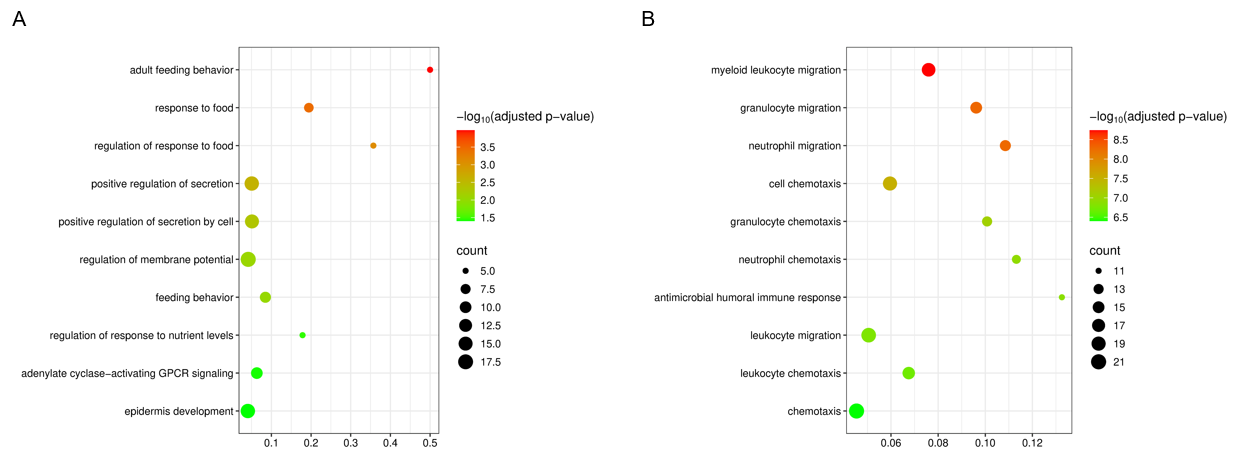


**Supplementary Figure 2.** Gene ontology enrichment analysis of the differentially expressed genes between (A) High- and low-TNB groups and (B) High- and low-TMB groups. The y-axis represents the significantly enriched BP terms, and the x-axis is the gene ratio.


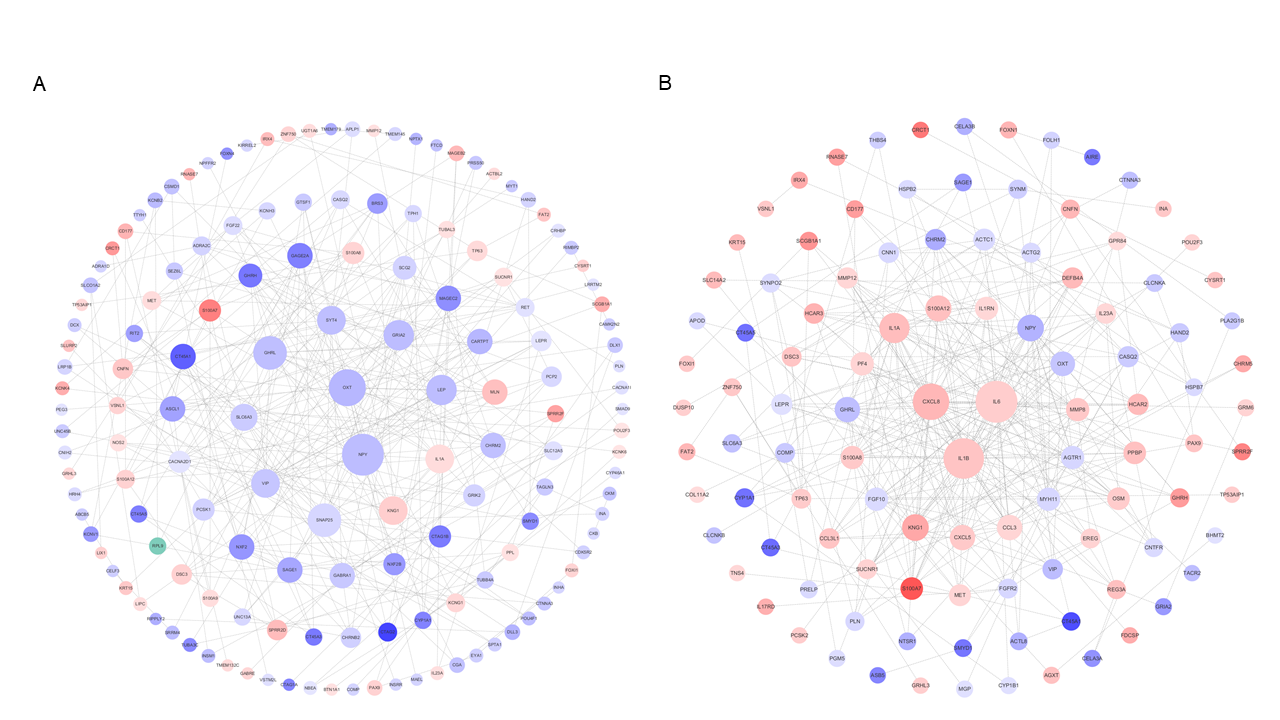


**Supplementary Figure 3.** Protein-protein interaction network of DEGs. (A) The overall network of TNB-associated DEGs. (B) The overall network of TMB-associated DEGs. Node size is proportional to its degree. Purple circles represent upregulated DEGs, and red circles represent downregulated DEGs.
